# Scarring of the C8-T1 roots with partial avulsion in situ in total obstetric brachial plexus palsy

**DOI:** 10.1007/s00238-017-1281-3

**Published:** 2017-02-17

**Authors:** Mohammad M. Al-Qattan, Amel A. F. El-Sayed

**Affiliations:** 1 Department of Surgery, King Saud University, P.O. Box 18097, Riyadh, 11415 Saudi Arabia; 2 Department of Obstetrics and Gynecology, King Saud University, Riyadh, Saudi Arabia

**Keywords:** Obstetric brachial plexus, Total palsy, Surgery

## Abstract

**Background:**

Primary exploration of the brachial plexus in infants with obstetric palsy may reveal scarring of the lower roots with evidence of partial avulsion-in-situ. As we have been treating this lesion by neurolysis only, we aimed to investigate the recovery of hand function following such approach.

**Methods:**

A series of 14 cases of total obstetric palsy with with evidence of partial avulsion-in-situ of the lower roots were included. All lesions were treated by neurolysis only (with no neurotization of the lower roots). Management of the injured upper roots was done by neurotization. Recovery was assessed as per our motor grading system.

**Results:**

After a minimum follow-up of 4 years, hand functional recovery was considered good in 7 patients and excellent in the remaining 7 patients.

**Conclusions:**

We highlight the scarring of lower roots with evidence of partial avulsion-in situ in obstetric palsy. We also document that neurolysis is an acceptable approach to such lesions.

Level of Evidence: Level IV, therapeutic study.

## Introduction

Several authors defined the various types of the brachial plexus lesion in infants with obstetric brachial plexus palsy (OBBP). Terzis and Kokkalis [1] graded each root as follows: 0, avulsion; 1, avulsion/rupture; 2, rupture; 3, rupture/traction; 4, traction; and 5, normal. Gilbert [2] and Al-Qattan [3] described three main lesions: avulsion (when there is no communication between the root and the foramen; and the dorsal root ganglion is seen outside the foramen), avulsion in situ (when the root is still attached to foramen with no visible dorsal root ganglion; however, the root is soft, pale, and does not respond to stimulation), and neuromas. Clarke et al. [4] stressed on differentiating three types of neuromas-in-continuity: well-, poorly-, and non-conducting neuromas-in-continuity.

During primary surgical exploration of the brachial plexus in infants with total OBBP, the senior author has noted that the C8-T1 roots were sometimes injured by an injury that could not be fitted into any of the above lesion types. In these cases, the C8-T1 roots and the proximal part of the lower trunk were scarred (epineurial scarring). Upon external neurolysis (epineurectomy) of the scarred roots, 20–50% of the fascicles of one or both roots locked soft and pale with no response to stimulation, indicating partial avulsion in situ. The remaining fascicles of the roots were not pale or soft, and upon intraoperative nerve stimulation, there was either no stimulation or poor stimulation of digital flexors and the intrinsic muscles of the hand. In the current paper, this type of lesion will be named “Scarred Roots with Partial Avulsion-in-situ (SR-PA).” Over the last two decades, the senior author has done only external neurolysis for this lesion.

The aim of this retrospective study was to study the motor recovery of the hand in a series of infants with total OBBP and SR-PA of the C8 and T1 roots, treated by external neurolysis. The outcome of such lesions has not been previously investigated in the literature, and we aimed to document if external neurolysis is an acceptable approach to these lesions.

## Methods

Ethical approval was obtained to conduct this retrospective study. The study included all infants with total OBBP with SR-PA of the C8 and T1 roots treated surgically by the senior author (between 1995 and 2012) by external neurolysis of the lower two roots/lower trunk. Management of the upper three roots varied depending on the type of injury to the C5-C7 roots. We excluded patients who had a postoperative follow-up of less than 4 years.

At our OBBP Center, motor assessment is done in data sheets as per the motor grading system shown in Table 1. The table also defines what we consider as a satisfactory functional outcome. We usually do not offer secondary surgery (such as tendon transfers or osteotomies) to patient with a satisfactory outcome. Data collection for the current study was done just prior to surgery and at final follow-up. We do not do routine MRI or neurophysiological testing in infants with OBBP, and hence, such data were not available. The percentages of satisfactory limb functions at final follow-up were then calculated.

**Table 1:** Motor assessment in total obstetric brachial plexus palsy

Function	Scoring or measurement of function	Definition of a satisfactory functional outcome
Shoulder abduction	Measured in degrees of shoulder abduction	Abduction 120° or more
Shoulder external rotation	1 = the hand reaches the abdomen or thorax; 2 = The hand reaches the mouth; 3 = The hand reaches the ear; 4 = The hand reaches the occiput; 5 = Normal external rotation.	A score of 3 or more
Elbow flexion & extension	0 = No motion; 1 = Active motion with gravity eliminated; 2 = Active motion against gravity, 3 = Active motion against resistance reaching ≤ ½ normal range, 4 = Active motion against resistance reaching > ½ normal range, 5 = Normal.	A score of 4 or 5
Forearm rotation	1 = Over pronated forearm causing functional or cosmetic disability, 2 = Over supinated forearm causing functional or cosmetic disability, 3 = functional forearm position (mid-pronation/supination or slight pronation) with no or minimal active motion, 4 = same as part 3 but good (over 20^o^) active pronation/ supination, 5 = Normal.	A score of 3 or more
Wrist extension	0 = Non-functional (No active extension or extension only with gravity eliminated), 1 = Active wrist extension to less than neutral, 2 = Active wrist extension to neutral or more than neutral, 3 = Normal wrist extension	A score of 2 or 3
Digital extension	0 = Non-functional (No active extension or extension only with gravity eliminated), 1 = Active digital extension to less than ½ range of motion, 2 = Active digital extension to more than ½ range of motion, 3 = Normal digital extension	A score of 2 or 3
Wrist flexion	0 = No active wrist flexion 1 = Any active wrist flexion 2 = Normal wrist flexion	A score of 1 or 2
Hand Function	0 = Useless hand: Complete paralysis or slight finger motion of no use, useless thumb 1 = Poor function: only very weak grip possible 2 = Fair function: there is some active flexion and extension the fingers and some thumb mobility but the hand posture is intrinsic minus. 3 = Good function: same as 2 but there is no intrinsic minus posture (intrinsic balance) 4 = Excellent function: near normal active finger flexion/extension and thumb mobility; with some active intrinsic function 5 = Normal function	A score of 3 or more

## Results

A total of 14 infants with total OBBP were included in the study. The presentation was cephalic and delivery was done vaginally at term in all cases. Horner syndrome was seen in five cases. Ipsilateral clavicular fractures were seen in two cases. The time of surgery ranged from 3 to 4 months (mean 3.5 months). Intraoperative findings revealed SR-PA of the C8-T1 roots in all cases. Details of intraoperative findings and the surgical management are shown in Table 2. As seen in the table, the SR-PA lesion was also seen at the C7 root in two cases, and this was treated by external neurolysis similar to the management of the lesion at the C8-T1 roots. Re-innervation of the supra/infraspinatus was done by a spinal accessory to suprascapular nerve transfer in cases # 1–12 and by intraplexus neurotization (from C5) in the cases # 13 and 14. Reconstruction of the upper/middle trunks was done using bilateral sural nerve grafts coapted between the ruptured upper roots proximally and the trunks distally. Fibrin glue was used for coaptation. The available upper roots for intraplexus neurotization varied from one to three roots. Regardless of the number of available upper roots, re-innervation of the biceps always took the priority with three cable grafts connected to the anterior division of the upper trunk.

**Table 2:** Intraoperative findings and surgical management in the study group (*N* = 14)

Case #*	Intraoperative findings	Operative management
1–4	C5: Rupture, C6-C7: Avulsion, C8-T1: SR-PA **	11th to SSN ***, C5 to upper / middle trunks, Neurolysis of C8 – T1.
5–10	C5 - C6: Rupture, C7: Avulsion, C8-T1: SR-PA	11th to SSN, C5 & C6 to upper / middle trunks, Neurolysis of C8 – T1.
11	C5 – C6: Rupture, C7: SR-PA, C8-T1: SR-PA	11th to SSN, C5 & C6 to upper trunk, Neurolysis of C7, C8, and T1.
12	C5: Rupture, C6: Avulsion, C7: SR-PA, C8-T1: SR-PA	11th to SSN, C5 to upper trunk, Neurolysis of C7, C8, and T1.
13–14	C5, C6 and C7: Rupture, C8-T1: SR-PA	C5, 6, 7 to SSN and upper / middle trunks, Neurolysis of C8 – T1.

*Case number: cases of similar findings were grouped together

**SR-PA: Scarred Root with Partial Avulsion-in-situ

***11th to SSN: Spinal accessory (11th Cranial Nerve) to suprascapular nerve transfer

Table 3 shows the pre-operative motor assessment. The clinical picture was consistent with a total palsy, and all patients had zero functions at the wrist, digits, and hand intrinsic muscles. Note should be given that our functional motor grading system does not take into consideration the motion of individual muscles. For example, none of the infants had any preoperative active motion in pronation or supination. Yet, all infants scored a grade of 3 for forearm rotation because the forearm was neither over-pronated or over-supinated (Table 3).

**Table 3:** Immediate pre-operative motor assessment in the study group (*N* = 14)

Pre-operative motor assessment as per the grading system shown in Table 1:									
Case Number	Sh. Ab	Sh. Ext Rot.	Elb. Flex.	Elb. Ext	Forearm Rot	Wrist Ext	Digit. Ext	Wrist Flex	Hand Function
1	0	1	0	2	3	0	0	0	0
2	0	1	0	2	3	0	0	0	0
3	20°	1							
4	10°	1	0	2	3	0	0	0	0
5	0	1	0	2	3	0	0	0	0
6	0	1	1	3	3	0	0	0	0
7	20°	1	0	2	3	0	0	0	0
8	10°	1	0	3	3	0	0	0	0
9	10°	1	0	2	3	0	0	0	0
10	0	1	0	3	3	0	0	0	0
11	0	1	1	3	3	0	0	0	0
12	0	1	0	2	3	0	0	0	0
13	10°	1	1	3	3	0	0	0	0
14	10°	1	1	3	3	0	0	0	0

*Sh. Ab.* shoulder abduction, *Sh. Ext. Rot.* shoulder external rotation, *Elb. Flex.* elbow flexion, *Elb. Ext.* elbow extension, *Forearm Rot.* forearm rotation, *Wrist Ext.* wrist extension, *Digit. Ext*. digital extension, *Wrist flex* wrist flexion

Follow-up ranged between 4 and 6 years (range 4.8 years). Table 4 shows the postoperative motor assessment at final follow-up prior to any secondary surgical procedures. The most important observation is that all patients recovered either an excellent or good hand function. Furthermore, the two patients (patients # 11 and 12) who also had SR-PA lesion of the C7 root recovered normal wrist/digital extension.

**Table 4:** Post-operative motor assessment at final follow up prior to any secondary procedures in the study group (*N* = 14)

Post-operative motor assessment as per the grading system shown in Table 1:									
Case Number	Sh. Ab	Sh. Ext Rot.	Elb. Flex.	Elb. Ext	Forearm Rot	Wrist Ext	Digit. Ext	Wrist Flex	Hand Function
1	50	3	4	4	3	0	1	2	3
2	60	2	3	4	3	1	2	2	3
3	40	3	5	4	3	1	3	1	3
4	120	3	3	4	3	0	1	2	4
5	110	4	5	4	4	2	3	2	4
6	130	1	5	4	3	3	3	2	3
7	120	1	5	4	4	3	2	2	4
8	110	1	5	4	3	2	2	1	4
9	160	4	5	4	3	3	3	2	3
10	90	1	5	4	3	2	3	1	3
11	170	4	5	4	4	3	3	2	3
12	160	4	4	4	3	3	3	2	4
13	140	4	5	4	4	3	3	2	4
14	150	4	5	4	4	3	3	2	4

*Sh. Ab.* shoulder abduction, *Sh. Ext. Rot.* shoulder external rotation, *Elb. Flex.* elbow flexion, *Elb. Ext.* elbow extension, *Forearm Rot.* forearm rotation, *Wrist Ext.* wrist extension, *Digit. Ext.* digital extension, *Wrist flex* wrist flexion

The percentages of satisfactory outcomes of various motor functions are shown in Table 5. The worst outcomes were seen in shoulder abduction and external rotation. Two patients had unsatisfactory elbow flexion. All patients had a mild degree (5–35°) of elbow flexion contracture limiting full elbow extension, but all patients still qualified for a satisfactory elbow extension function. None of the patients had an over-pronated or over-supinated forearm (hence, the 100% satisfactory outcome for forearm rotation). Wrist and digital extension was satisfactory in 71.4 and 85.7%, respectively. All patients had active wrist flexion and a satisfactory hand function as mentioned above.

**Table 5:** The % of satisfactory outcome of various motor functions in the study group (*N* = 14)

Motor function	Number of patients with a satisfactory outcome^a^	% Of patients with a satisfactory outcome^a^
Shoulder abduction	8	57.1%
Shoulder external rotation	9	64.3%
Elbow flexion	12	85.7%
Elbow extension	14	100%
Forearm rotation	14	100%
Wrist extension	10	71.4%
Digital extension	12	85.7%
Wrist flexion	14	100%
Hand function	14	100%

^a^The definitions of satisfactory outcomes are shown in Table 1

## Discussion

The current article highlights the SR-PA lesion of the lower two roots of the brachial plexus in infants with total OBBP and investigates the outcome of hand function 4 years following neurolysis. The injured lower roots had no bulge, and hence, the lesion was not a neuroma-in-continuity. The dorsal root ganglion was not visible, and hence, it could be considered as total avulsion. Upon initial inspection, the lower roots were scarred. Following neurolysis, there was no evidence of rupture. However, part of the root was pale, soft, and did not respond to stimulation. This indicated either partial intra-foraminal rupture or partial avulsion in situ. Avulsion in situ has only been described in the literature to affect the entire and not part of the root. Avulsion in situ is commonly seen in the upper roots following breech deliveries [2, 3]. Al-Qattan [3] reported on the outcome of conservative management of avulsion in situ in 7 roots (all involving upper roots) in infants with OBBP. Excellent spontaneous recovery was seen in 5 out of the 7 roots. Gilbert [2] reported on 26 OBBP cases with avulsion in situ of the upper roots treated conservatively and reported excellent spontaneous recovery in 50% of cases. One can afford to try conservative management of avulsion in situ of the upper roots because of the proximity of affected muscles. Gilbert [2] stressed that if shoulder or elbow function does not recover by 1 year of age following the primary conservative management, intraplexus neurotization is done for salvage in a secondary procedure around 1 year of age. Currently, the biceps may also be salvaged at 1 year of age using a distal nerve transfer [5, 6]. The conservative approach for total avulsion in situ of the lower roots is not advised since the time needed to check recovery is beyond salvage by a secondary neurotization.

The most important finding of the current study is the excellent/good hand recovery in all patients following neurolysis of the SR-PA lesion of the lower roots. In our series, 50% had excellent and the other 50% had good hand functional recovery. This may indicate that the recovery of the partial avulsion in situ has only occurred in the former group of patients. Furthermore, excellent recovery of the SR-PA lesion of the C7 root was also observed in patients 13 and 14. Hence, neurolysis of SR-PA lesion should be considered an acceptable approach.

The main strength of the current study is the inclusion of a specific group of infants with total OBBP treated in a uniform manner by the same surgeon, with documentation of function according to a sound motor evaluation system. However, several weak points should be highlighted. The definitions of “root avulsion” and “root avulsion in situ” are well described in the literature. However, from the clinical point of view, these definitions are just hypothetical; as the absence of ganglion in the surgical field allows no definitive conclusion, neither does the absence of a motor stimulation response of the lower trunk at 3 months of age. Another weak point is the lack of MRI in our series. However, the accuracy of MRI to diagnose root avulsion is not 100%. On the other hand, Hashimoto et al. [7] were able to differentiate between complete versus incomplete avulsion root avulsion by MRI in infants with OBBP. Intra-operative somatosensory evoked potential would be another useful tool to diagnose complete root avulsion. However, the test does not discriminate incomplete root avulsion from intact roots [7].

Primary reconstruction of the brachial plexus is usually performed by experienced surgeons in the field. The partial avulsion in situ in the current series was diagnosed by observation (the color and consistency of the root not being uniform) as well as by dissection of the root at the foramen level to ensure that there was no element of axonal rupture. The presence of partial avulsion also explains the satisfactory recovery of the C8-T1 lesion in our series.

The result of neurotization of the upper part of the plexus in the current series is consistent with other series in the literature [8–10]. The results at the shoulder are known to be inferior to the results at the elbow, and this is probably multi-factorial [11]. The surgeon generally gives more priority (i.e., more cable graft) to innervate the biceps. Furthermore, the suprascapular nerve may have a second area of scarring at the supra-scapular notch [11]. Another major factor affecting the results is the number of roots available for intraplexus neurotization of the upper/middle trunks [8]. In our series, cases # 1–4 had C5 rupture and C6–7 avulsion. In contrast, cases # 13 and 14 had rupture of C5, 6, and 7 roots (Table 2). As expected, the recovery at the shoulder/elbow/wrist was better in the latter two patients since all 3 ruptured roots were available for intraplexus neurotization (Table 4).

Another interesting point is the presence of Horner syndrome in only 5 of the patients. The surgical literature on OBBP only includes Horner syndrome in a subset of infants with total palsy [12] and documents the syndrome as a poor prognostic sign for satisfactory motor recovery [13]. However, the neurology literature documents that the syndrome is not always associated with total palsy, as it is occasionally seen in extended Erb birth palsy [14]. In fresh cadaveric dissections, the origin of the white rami communicantes of the stellate ganglion is from the C7, C8, and T1 spinal nerves in 87% of infant cadavers and from C8/T1 roots in 100% of adult cadavers [15]. This also explains the lack of Horner syndrome in some infants with T1 root complete avulsion. In our series, only parts of the lower roots had partial avulsion, explaining the absence of Horner in 9 out of 14 infants.

Finally, it is important to realize that scientific analysis of the surgical findings and outcome is not easy in OBBP, and currently, there are no randomized controlled studies on the topic [16]. The entity we describe in the current study is only seen in a small group of infants with OBBP, but the decision of leaving or neurotizing such a C8-T1 lesion has always been difficult. Our main aim was to document the outcome following neurolysis of this lesion.

In conclusion, we highlight the SR-PA lesion of the lower roots of the brachial plexus in OBBP. We also document that neuralysis is an acceptable approach to such lesions.
