# Erratum to: Scarring of the C8-T1 roots with partial avulsion in situ in total obstetric brachial plexus palsy

**DOI:** 10.1007/s00238-017-1331-x

**Published:** 2017-05-25

**Authors:** Mohammad M. Al-Qattan, Amel A. F. El-Sayed

**Affiliations:** 1 Department of Surgery, King Saud University, P.O. Box 18097, Riyadh, 11415 Saudi Arabia; 2 Department of Obstetrics and Gynecology, King Saud University, Riyadh, Saudi Arabia

**Erratum to: European Journal of Plastic Surgery**

**DOI 10.1007/s00238-017-1281-3**

The article Scarring of the C8-T1 roots with partial avulsion in situ in total obstetric brachial plexus palsy, written by Mohammad M. Al-Qattan & Amel A.F El-Sayed, was originally published electronically on the publisher’s internet portal (currently SpringerLink) on 17 February 2017 without open access.

With the author(s)’ decision to opt for Open Choice the copyright of the article changed on 24 May 2017 to © The Author(s) 2017 and the article is forthwith distributed under the terms of the Creative Commons Attribution 4.0 International License (http://creativecommons.org/licenses/by/4.0/), which permits use, duplication, adaptation, distribution and reproduction in any medium or format, as long as you give appropriate credit to the original author(s) and the source, provide a link to the Creative Commons license and indicate if changes were made.

The original article was corrected.

